# Local hyperthermia mediated by gold nanoparticle-integrated silicone-covered stent: feasibility and tissue response in a rat esophageal model

**DOI:** 10.1186/s41747-024-00438-0

**Published:** 2024-04-03

**Authors:** Jiaywei Tsauo, Yue Liu, Xiaowu Zhang, Yan Fu, He Zhao, Tao Gong, Jingui Li, Xiao Li

**Affiliations:** 1 Department of Interventional Therapy, National Cancer Center/National Clinical Research Center for Cancer/Cancer Hospital, Chinese Academy of Medical Sciences and Peking Union Medical College, Beijing, 100021 China; 2 Department of Interventional Radiology, Guangdong Provincial People’s Hospital (Guangdong Academy of Medical Sciences), Southern Medical University, Guangzhou, Guangdong 510080 China; 3 Department of Etiology and Carcinogenesis and State Key Laboratory of Molecular Oncology. National Cancer Center/National Clinical Research Center for Cancer/Cancer Hospital, Chinese Academy of Medical Sciences and Peking Union Medical College, Beijing, 100021 China

**Keywords:** Biliary tract, Gastrointestinal tract, Hyperthermia (induced), Neoplasms, Self-expandable metallic stents

## Abstract

**Background:**

To assess the feasibility and tissue response of using a gold nanoparticle (AuNP)-integrated silicone-covered self-expandable metal stent (SEMS) for local hyperthermia in a rat esophageal model.

**Methods:**

The study involved 42 Sprague–Dawley rats. Initially, 6 animals were subjected to near-infrared (NIR) laser irradiation (power output from 0.2 to 2.4 W) to assess the *in vitro* heating characteristics of the AuNP-integrated SEMS immediately after its placement. The surface temperature of the stented esophagus was then measured using an infrared thermal camera before euthanizing the animals. Subsequently, the remaining 36 animals were randomly divided into 4 groups of 9 each. Groups A and B received AuNP-integrated SEMS, while groups C and D received conventional SEMS. On day 14, groups A and C underwent NIR laser irradiation at a power output of 1.6 W for 2 min. By days 15 (3 animals per group) or 28 (6 animals per group), all groups were euthanized for gross, histological, and immunohistochemical analysis.

**Results:**

Under NIR laser irradiation, the surface temperature of the stented esophagus quickly increased to a steady-state level. The surface temperature of the stented esophagus increased proportionally with power outputs, being 47.3 ± 1.4 °C (mean ± standard deviation) at 1.6 W. Only group A attained full circumferential heating through all layers, from the epithelium to the *muscularis propria*, demonstrating marked apoptosis in these layers without noticeable necroptosis.

**Conclusions:**

Local hyperthermia using the AuNP-integrated silicone-covered SEMS was feasible and induced cell death through apoptosis in a rat esophageal model.

**Relevance statement:**

A gold nanoparticle-integrated silicone-covered self-expanding metal stent has been developed to mediate local hyperthermia. This approach holds potential for irreversibly damaging cancer cells, improving the sensitivity of cancer cells to therapies, and triggering systemic anticancer immune responses.

**Key points:**

• A gold nanoparticle-integrated silicone-covered self-expanding metal stent was placed in the rat esophagus.

• Upon near-infrared laser irradiation, this stent quickly increased the temperature of the stented esophagus.

• Local hyperthermia using this stent was feasible and resulted in cell death through apoptosis.

**Graphical Abstract:**

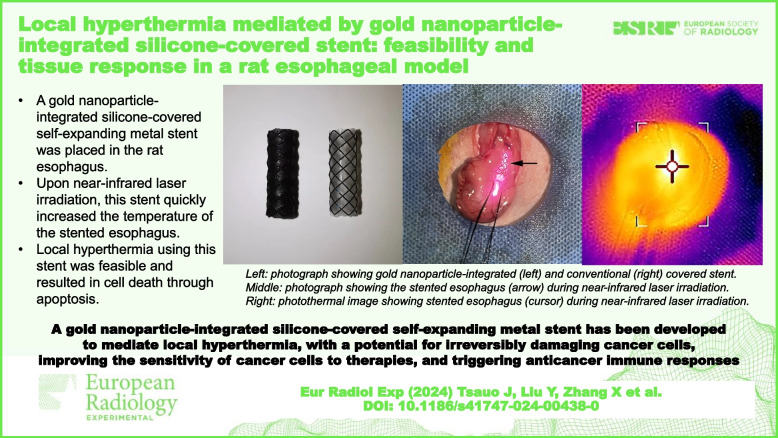

## Background

Self-expandable metal stent (SEMS) placement is a well-established method for the palliative treatment of malignant esophageal strictures [1]. This procedure quickly relieves dysphagia compared with brachytherapy and radiotherapy but does not treat the underlying cancer [2]. In addition, recurrent dysphagia occurs in approximately 14% of patients after SEMS placement because of tumor growth [3]. To address these limitations, a radioactive SEMS was introduced [4, 5]. In a randomized trial, this radioactive SEMS prolonged patient survival compared with a conventional SEMS [5]. Although further studies are necessary to confirm this result, the potential for local cancer therapy with SEMSs has been realized.

Recently, a gold nanoparticle (AuNP)-coated uncovered SEMS was investigated for its ability to mediate local heat treatment upon near-infrared (NIR) laser irradiation in a rat esophageal and gastric outlet model [6, 7]. This SEMS is of clinical interest because hyperthermia can induce irreversible damage to cancer cells, enhance the delivery of anticancer drugs, improve the sensitivity of cancer cells to therapies, and trigger systemic anticancer immune responses [8]. However, uncovered SEMSs are not recommended for patients with malignant esophageal strictures, as they are associated with a higher rate of tumor or tissue ingrowth compared to covered SEMSs [1].

Silicone is commonly used as the covering membrane for covered SEMSs due to its high chemical and thermal stability [9]. The dipping method is often employed to apply this silicone membrane to SEMSs [10]. By adding silver particles to the silicone solution before dipping, Lee et al. [11] successfully fabricated a silver particle-integrated silicone-covered SEMS for biliary obstructions. Using the same method, we fabricated an AuNP-integrated silicone-covered SEMS. The aim of this study was to evaluate the feasibility and tissue response of using this AuNP-integrated silicone-covered SEMS for local hyperthermia in a rat esophageal model.

## Methods

This study was approved by the Animal Research Committee of the National Cancer Center (identifier: NCC2019A114) on March 1, 2019, and conformed to the guidelines for the Care and Use of Laboratory Animals.

### SEMS description

**Fig. 1:**
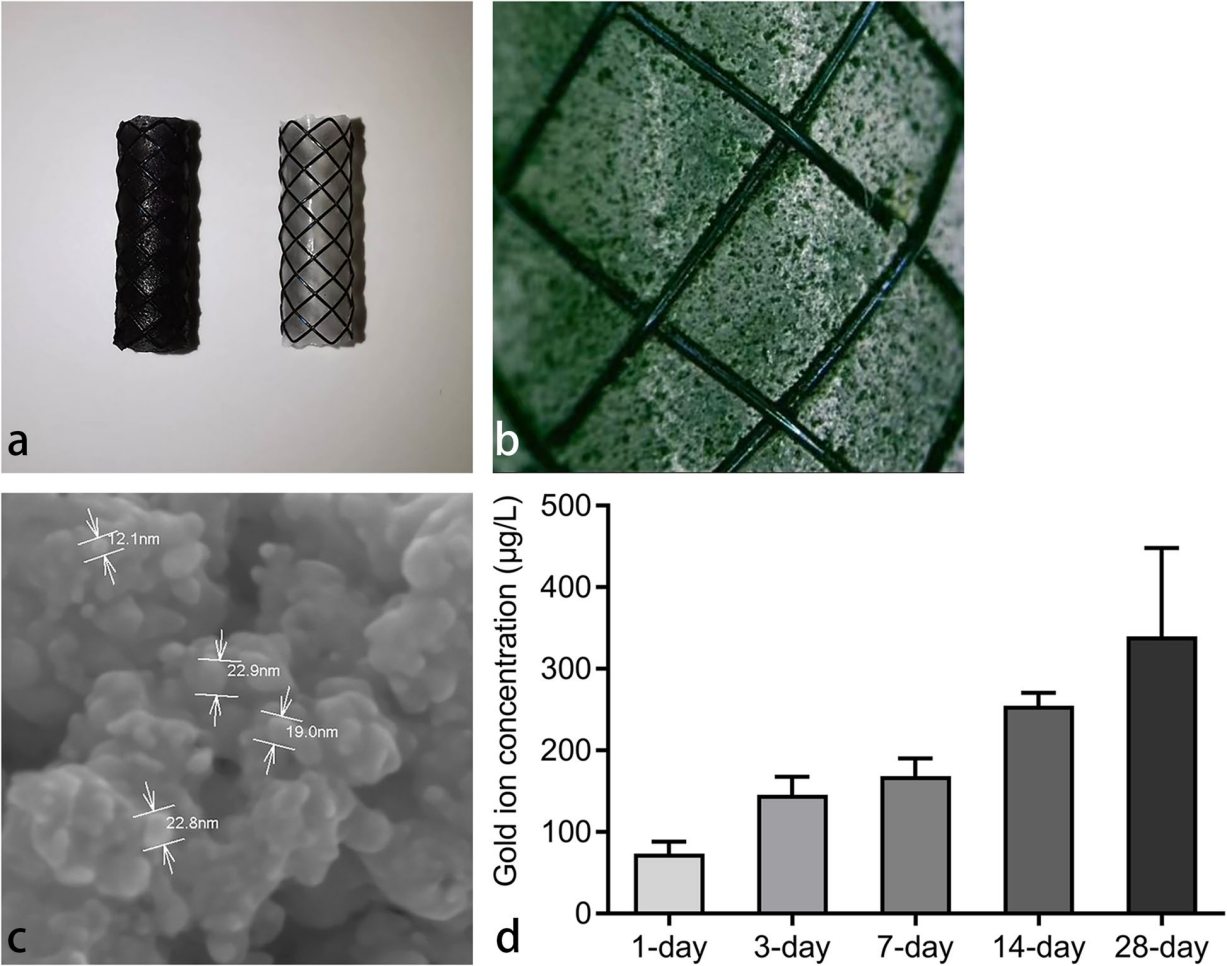
**a** Photograph showing the AuNP-integrated silicone-covered SEMS (left) and the conventional silicone-covered SEMS (right). **b** Light microscope image showing that the AuNPs were integrated into the silicone membrane of the SEMS. **c** Scanning electron microscope image showing the gold nanospheres with an average diameter of 20 nm. **d** Bar graph showing the amount of gold ions released from the AuNP-integrated silicone-covered SEMSs into phosphate-buffered saline at 37 °C over a span of 28 days. *AuNP*, Gold nanoparticle; *SEMS*, Self-expandable metal stent

The AuNP-integrated and conventional silicone-covered SEMSs used in this study were both supplied by Youan Medical (Beijing, China) (Fig. 1a,b). These SEMSs are braided from nitinol wire and are available in diameters ranging from 4 to 20 mm and lengths of 1 to 15 cm. Given the diameter and length of the rat esophagus, SEMSs with a 5 mm diameter and 1.5 cm length were selected for this study [12]. Both type of SEMSs were fully covered with a silicone membrane using the dipping method. Specifically, an 8 mm diameter cylindrical Teflon mandrel was inserted into the lumen of the SEMS and heat-treated at approximately 150 °C for around 2 h after being dipped in the silicone solution. To integrate AuNPs into the silicone membrane of the AuNP-integrated SEMS, gold nanospheres were added to the dipping solution at a concentration of 1.5 mg/mL. These gold nanospheres were produced using laser ablation and had an average diameter of 20 nm (Fig. 1c). Gold ion quantification in three AuNP-integrated SEMS samples was performed using inductively coupled plasma optical emission spectroscopy (iCAP7200; Thermo Fisher Scientific, Freemont, CA, USA). Each sample was immersed in aqua regia, heated to 180 °C for 1 h, and then the resulting solution’s gold ions were analyzed. Analysis revealed that the SEMSs contained a gold ion concentration of 115,997.2 ± 554.1 mg/kg (mean ± standard deviation). Gold ion release from AuNP-integrated SEMSs was quantified using inductively coupled plasma mass spectroscopy (NexION 300D; PerkinElmer, Shelton, CT, USA). Three AuNP-integrated SEMS samples were immersed in phosphate-buffered saline for 28 days at 37 °C, with gold ion concentrations in the saline analyzed at set intervals (days 1, 3, 7, 14, and 28) over a 28-day period. Analysis indicated minimal gold ion release from the SEMSs over the 28-day period (Fig. 1d).

### *In vitro* heating characteristics

To evaluate the *in vitro* heating characteristics of the SEMSs used in this study, we irradiated them with an 808 nm NIR laser (Lumen Photonics, Beijing, China) at power outputs ranging from 0.2 to 0.8 W using a 1 mm-diameter optic fiber (Lumen Photonics, Beijing, China). The surface temperature of these SEMSs was then measured using an infrared thermal camera (E5-XT; Teledyne FLIR, OR, USA). Under NIR laser irradiation, the surface temperature of the AuNP-integrated SEMSs quickly increased and stabilized, while the conventional SEMSs experienced a slower increase, requiring ≥ 30 s to reach a steady-state temperature. After the cessation of NIR laser irradiation, the surface temperature of all SEMSs quickly returned to room temperature. The *in vitro* steady-state surface temperatures of the SEMSs are summarized in Table 1. For both SEMS types, the surface temperature increased proportionally with power outputs. However, the AuNP-integrated SEMSs consistently showed notably higher temperatures compared to conventional SEMSs.

**Table 1:** *In vitro* steady-state surface temperatures of SEMSs under NIR laser irradiation

Power output (W)	Steady-state surface temperature (°C)	
	AuNP-integrated SEMS (*n* = 24)	Conventional SEMS (*n* = 18)
0.2	35.9 ± 0.3	24.4 ± 1.5
0.4	43.9 ± 0.4	30.1 ± 1.0
0.6	53.5 ± 0.6	36.3 ± 1.0
0.8	62.9 ± 2.1	41.1 ± 2.1

Data are given as mean ± standard deviation. *AuNP* Gold nanoparticle, *NIR* Near-infrared, *SEMS* Self-expandable metal stent

### *In vivo* heating characteristics

**Fig. 2:**
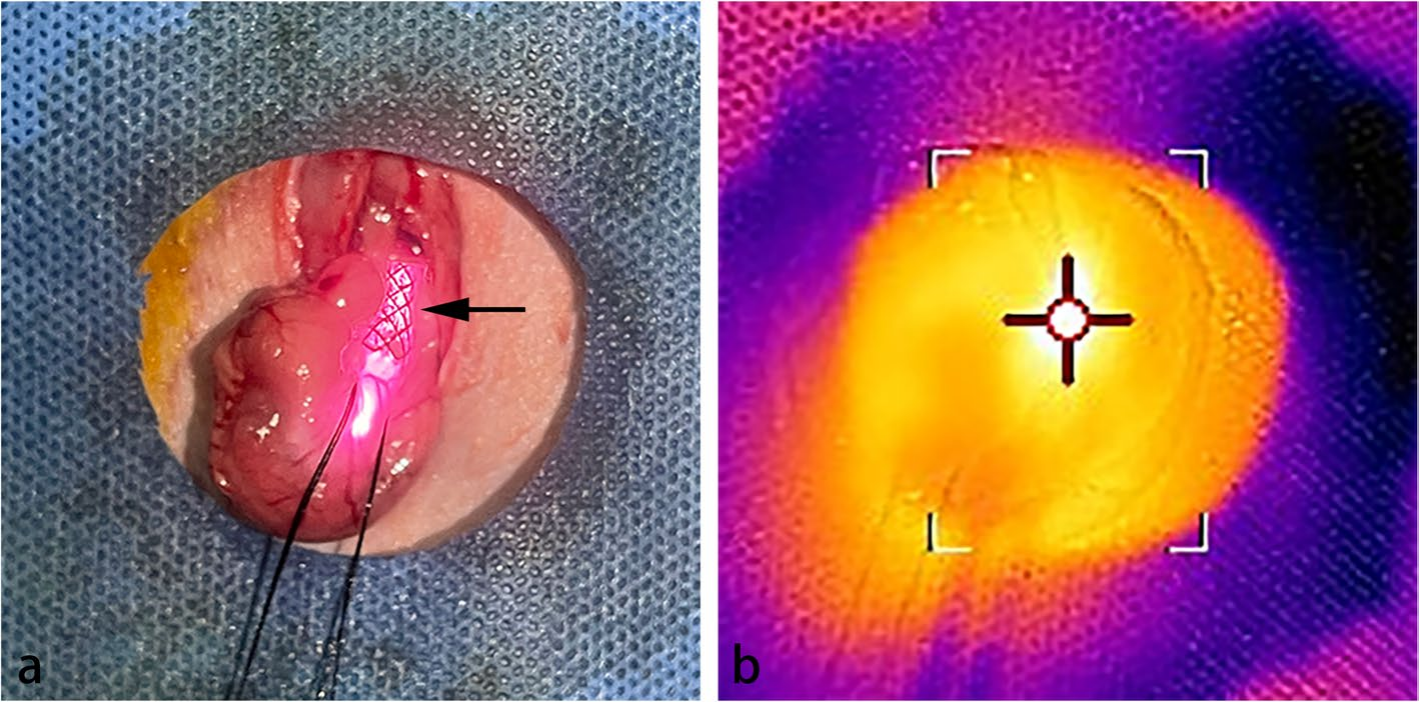
**a** Photograph showing the stented (AuNP-integrated silicone-covered SEMS) esophagus (arrow) during NIR laser irradiation. **b** Photothermal image showing the stented (AuNP-integrated silicone-covered SEMS) esophagus (cursor) during NIR laser irradiation. *AuNP*, Gold nanoparticle; *NIR*, Near-infrared; *SEMS*, Self-expandable metal stent

**Fig. 3:**
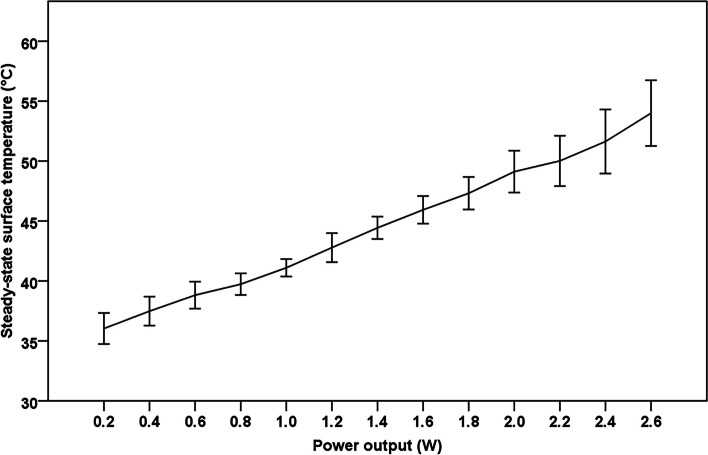
Line diagram showing the mean *in vivo* steady-state surface temperatures of the stented (AuNP-integrated silicone-covered SEMS) esophagus with NIR laser irradiation. Error bars are standard deviations. *AuNP*, Gold nanoparticle; *NIR*, Near-infrared; *SEMS*, Self-expandable metal stent

Six male Sprague–Dawley rats, weighing between 300 and 350 g, were used to assess the *in vitro* heating characteristics of the AuNP-integrated SEMS. After AuNP-integrated SEMS placement, these animals were immediately exposed to 808 nm NIR laser (Lumen Photonics, Beijing, China) with power outputs ranging from 0.2 to 2.4 W. A 1 mm-diameter optic fiber (Lumen Photonics, Beijing, China) was used for the irradiation, and it was inserted into the stented esophagus through a 6-Fr sheath (constructed in-house) (Fig. 2a). The surface temperature of the stented esophagus was monitored using an infrared thermal camera (E5-XT; Teledyne FLIR, OR, USA). Upon NIR laser irradiation, the surface temperature quickly increased to a steady-state level and then promptly returned to core body temperature after the irradiation ceased (Fig. 2b). The steady-state surface temperature of the stented esophagus increased proportionally with the power outputs, ranging from 37.5 ± 1.2 °C (mean ± standard deviation) at 0.2 W to 54.0 ± 2.7 °C at 2.4 W (Fig. 3). At a power output of 1.6 W, the steady-state surface temperature was 47.3 ± 1.4 °C. The cumulative equivalent min at 43 °C (CEM43) is a metric commonly utilized in hyperthermia research to standardize the thermal dose [13]. At 47.3 °C for 2 min, the CEM43 value is approximately 39.4 min.

### Animal study

**Fig. 4:**
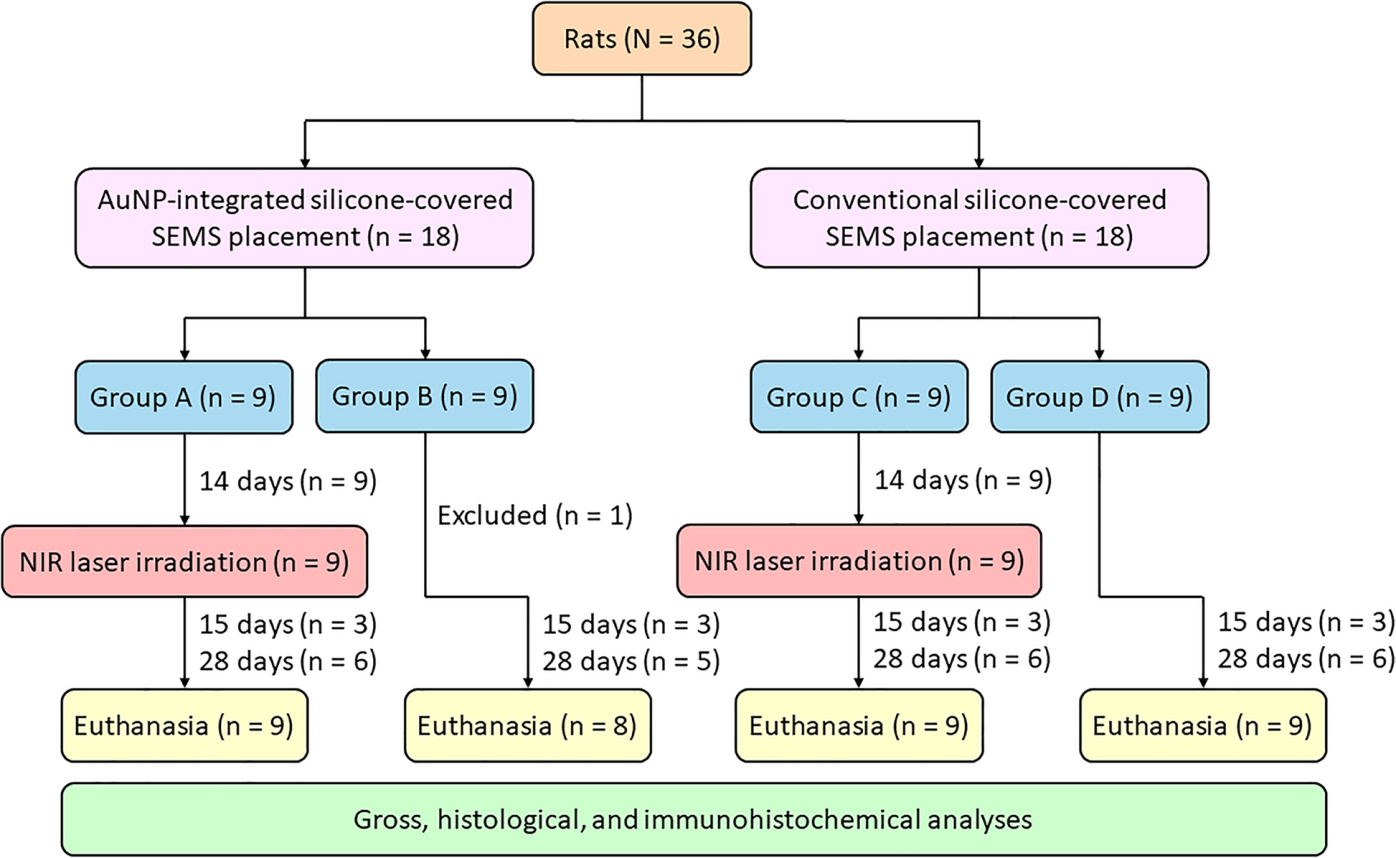
Flow diagram showing the study design and the number of rats that completed each part of the study. *AuNP*, Gold nanoparticle; *NIR*, Near-infrared; *SEMS*, Self-expandable metal stent

Thirty-six male Sprague–Dawley rats, weighing between 300 and 350 g, were randomly divided into four groups using computer-generated random numbers (Fig. 4). Animals in groups A (*n* = 9) and B (*n* = 9) received AuNP-integrated SEMS placement, while those in groups C (*n* = 9) and D (*n* = 9) received conventional SEMS placement. Fourteen days after SEMS placement, animals in groups A and C underwent peroral NIR laser irradiation at a power output of 1.6 W for 2 min. In contrast, animals in groups B and D did not undergo NIR laser irradiation. This experimental design was intended to confirm whether the tissue response was specifically induced by the AuNP-integrated SEMS under near-infrared laser irradiation. Three animals from each group were euthanized 15 days after SEMS placement. The remaining animals were euthanized 28 days after SEMS placement. All animals were euthanized using a gradual-fill method of carbon dioxide inhalation to ensure minimal distress. The concentration of carbon dioxide was progressively increased to induce unconsciousness and subsequently death. After the animals were euthanized, gross, histological, and immunohistochemical analyses were conducted.

### SEMS placement procedure

After 8 h of fasting, the rats were anesthetized with an intramuscular injection of a mixture of 50 mg/kg zolazepam and tiletamine. A 2 cm incision along the midline of the upper abdomen was made to expose the lower esophagus. A 6-Fr sheath (constructed in-house) loaded with a SEMS was inserted into the lower esophagus through the mouth. Under direct visualization through the semitransparent esophageal wall, the SEMS was placed in the lower esophagus. To prevent stent migration, the SEMS was sutured to the lower esophageal wall at three points using 4–0 silk sutures. The abdominal cavity was then closed with 4–0 Vicryl and silk sutures. After SEMS placement, radiographs were taken at 14 and 28 days to monitor stent migrations.

### Local hyperthermia procedure

**Fig. 5:**
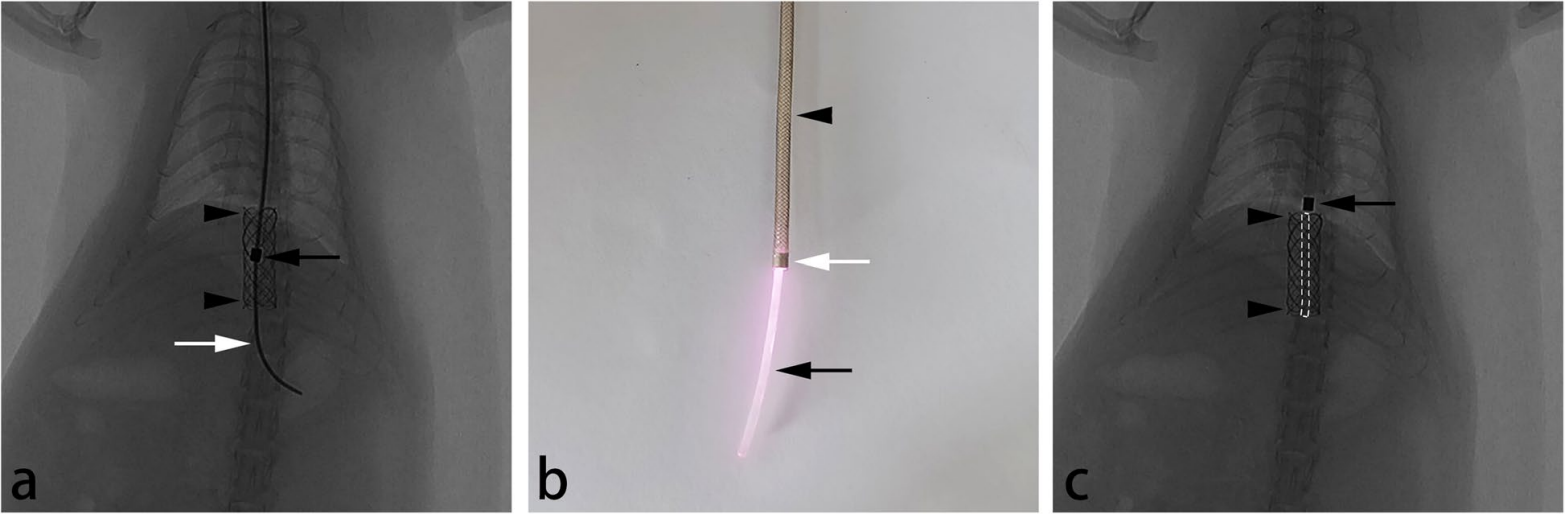
**a** Fluoroscopic image showing that the 5-Fr sheath (black arrow) was inserted in the stented (AuNP-integrated silicone-covered SEMS) esophagus (arrowhead) over a 0.035-in guidewire (white arrow). **b** Photograph showing the 1-mm-diameter optic fiber (black arrow) which was inserted through the 5-Fr sheath (arrowhead) with a radiopaque tip (white arrow). **c** Fluoroscopic image showing that the 5-Fr sheath (arrow) was withdrawn from the stented (AuNP-integrated silicone-covered SEMS) esophagus (arrowhead) with the 1-mm-diameter optic fiber (not visible fluoroscopically) left in place. Dotted line indicates the presumed location of the optic fiber. *AuNP*, Gold nanoparticle; *NIR*, Near-infrared; *SEMS*, Self-expandable metal stent

After fasting for 8 h, the rats were anesthetized with an intramuscular injection of a 50 mg/kg mixture of zolazepam and tiletamine. Under fluoroscopic guidance, a 5-Fr sheath with a radiopaque tip (constructed in-house) was inserted through the mouth into the stented esophagus over a 0.035 inch guidewire (Radiofocus; Terumo, Tokyo, Japan) (Fig. 5a). A 1 mm-diameter optic fiber (Lumen Photonics, Beijing, China) was then inserted through the sheath until the tip of the optic fiber reached the tip of the sheath. The sheath was then withdrawn from the stented esophagus, leaving the optic fiber in place, and 808 nm NIR laser irradiation (Lumen Photonics, Beijing, China) was performed at a power output of 1.6 W for 2 min (Fig. 5b,c).

### Histological and immunohistochemical analyses

The stented esophagus was harvested from rats euthanized either 15 or 28 days after SEMS placement. The tissue samples, once fixed and embedded in paraffin, were sectioned into 4 μm slices and stained with hematoxylin and eosin for histological analysis. For immunohistochemical analysis, sections from animals euthanized at 15 days after SEMS placement were deparaffinized, rehydrated, and incubated with a primary antibody against heat shock protein 70 (HSP70) (ab2747; Abcam, Cambridge, UK; diluted 1:400). Conversely, sections from 28-day euthanized animals underwent similar IHC preparation and were stained using terminal deoxynucleotidyl transferase-mediated dUTP nick end labeling (TUNEL) (ApopTag; Qbiogene, Darmstadt, Germany) and a primary antibody against Receptor-interacting kinase 3 (RIP3) (sc-374639; Santa Cruz Biotechnology, CA, USA; diluted 1:50). After staining, sections were examined using a digital slide scanner (Aperio ScanScope CS; Leica Biosystems, CA, USA). Epithelial and submucosal layer thickness and percentages of TUNEL-, HSP70-, and RIP3-positive cells were assessed using ImageJ v1.53 (NIH, MD, USA). Inflammatory cell infiltration was graded from 1 (mild) to 5 (severe). Data were averaged from 2 segments (proximal and distal) and 6 points on each segment’s circumference. Two blinded observers reached consensus on results.

### Statistical analysis

For normally distributed data, one-way ANOVA was used to determine if there were statistically significant differences among the groups. In cases where the assumption of equal variances was violated, Welch’s ANOVA was used instead. In instances where the ANOVA indicated statistically significant differences, Tukey’s HSD pairwise comparison was used to identify specific group differences. Non-normally distributed data were evaluated using the Kruskal–Wallis test, followed by Dunn-Bonferroni post hoc tests for statistically significant findings. A *p* value of < 0.05 denoted statistical significance. All statistical analyses were conducted with SPSS Statistics v21.0 (IBM Corp., Armonk, NY, USA).

## Results

### Technical success and adverse events

SEMS placement was successfully achieved in all 36 rats without any procedure-related adverse events (AEs). Similarly, NIR laser irradiation was successfully performed in all 18 animals from groups A and C, with no procedure-related AEs. Fourteen days after SEMS placement, a radiograph revealed stent migration into the stomach in one animal from group B. This animal was subsequently euthanized and excluded from the analysis. The remaining 35 animals survived until the end of the experimental period. However, 28 days after SEMS placement, radiographs detected stent migration into the stomach in 18 animals. This included 6 animals in group A, 3 in group B, 5 in group C, and 4 in group D. These 18 animals were not excluded from the analysis, as the stent migration happened after the NIR laser irradiation had been performed in groups A and C. Gross examination revealed no abnormalities, such as ulceration, perforation, or hemorrhage, in the lower esophagus or surrounding organs of any animal. The SEMS was successfully extracted and found intact in each animal.

### Histological analysis

**Fig. 6:**
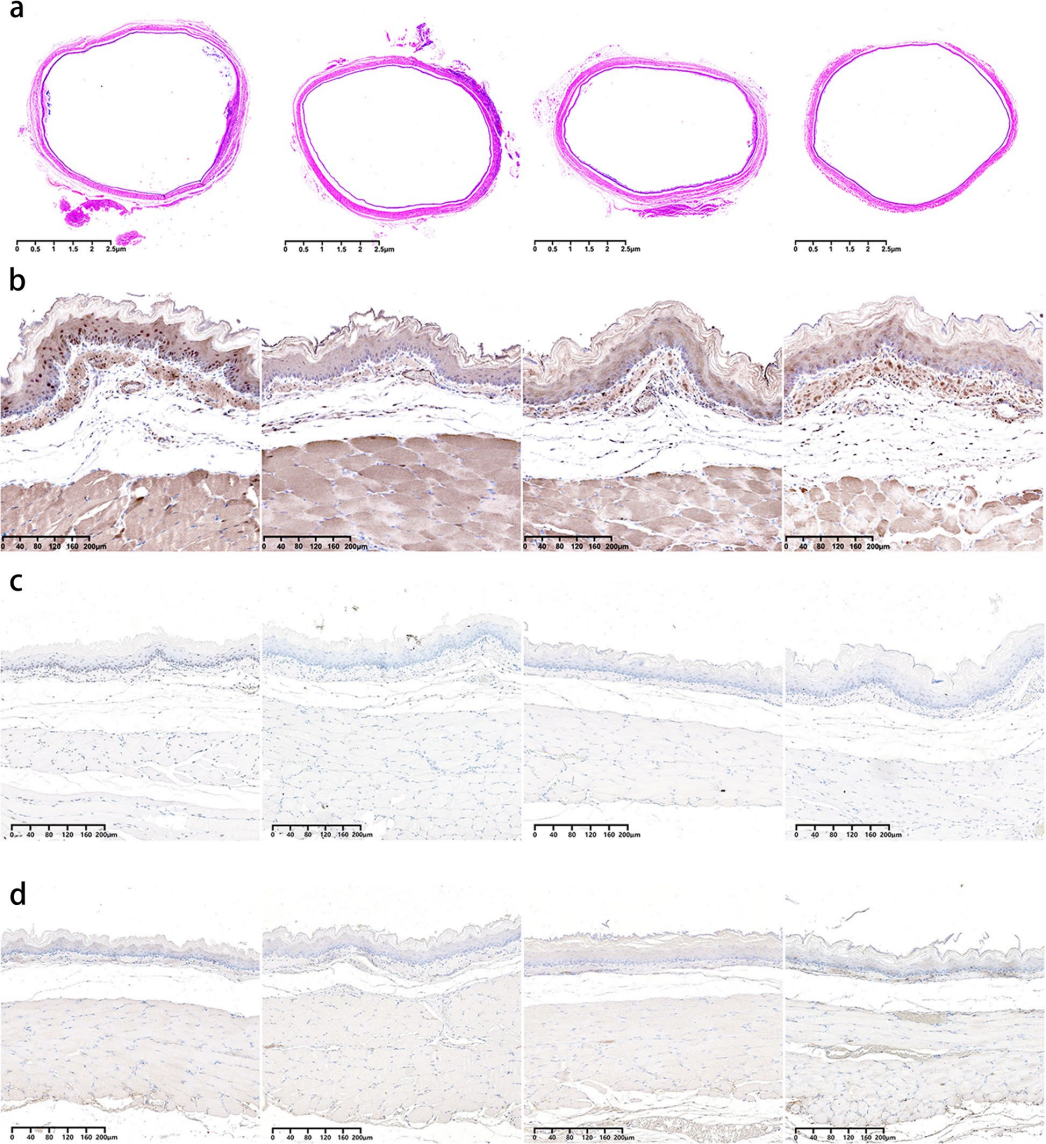
**a** Hematoxylin and eosin-stained representative histological images of the stented esophagus (× 1 magnification). From left to right: groups A through D. **b** HSP70-stained representative histochemical images of the stented esophagus (× 10 magnification). From left to right: groups A through D. **c** TUNEL-stained representative histochemical images of the stented esophagus (× 10 magnification). From left to right: groups A through D. **d** RIP3-stained representative histochemical images of the stented esophagus (× 10 magnification). From left to right: groups A through D. *HSP70*, Heat shock protein 70; *RIP3*, Receptor-interacting kinase 3; *TUNEL*, Terminal deoxynucleotidyl transferase-mediated dUTP nick end labeling

**Fig. 7:**
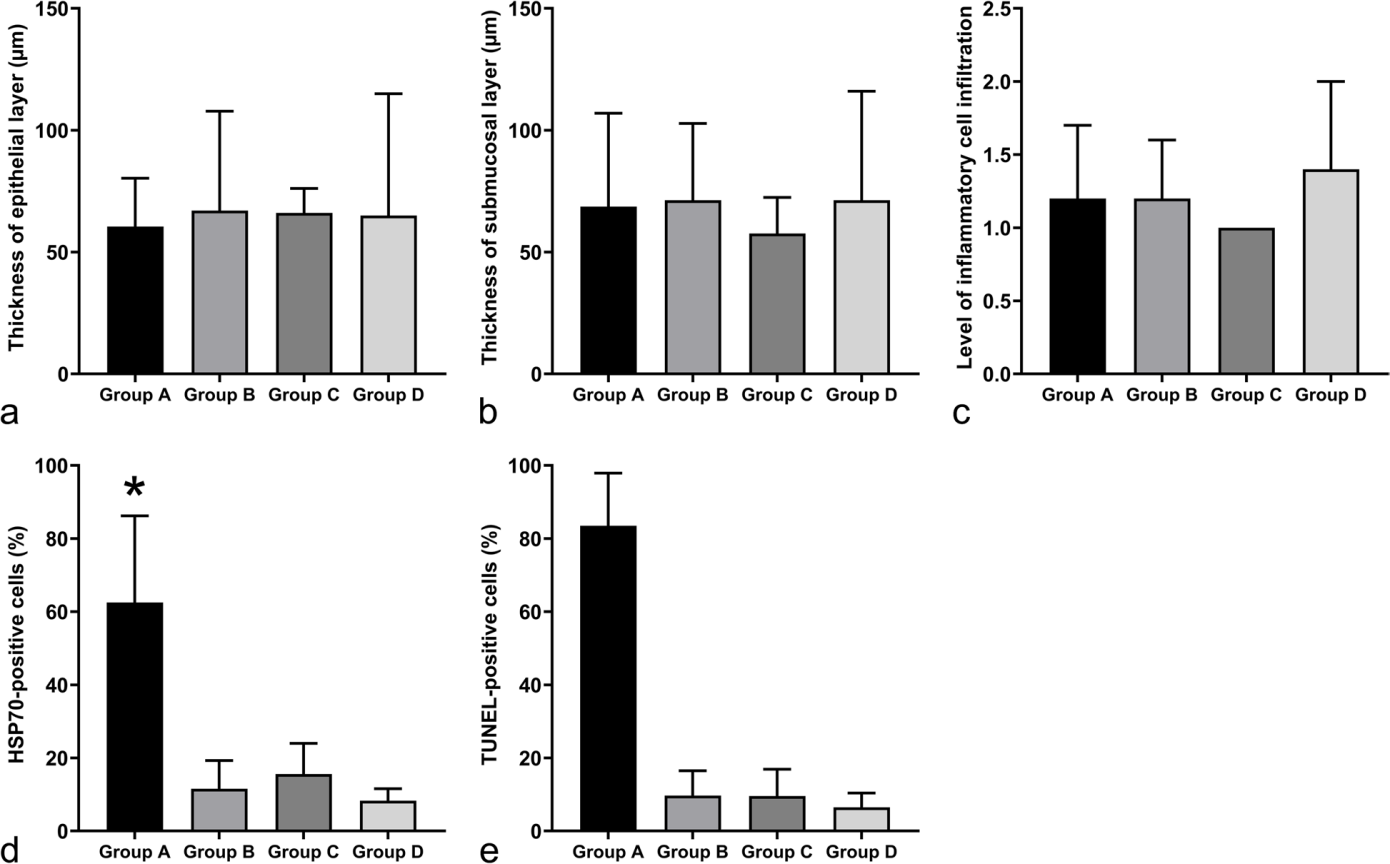
**a** The thickness of epithelial layer. **b** The thickness of submucosal layer. **c** The level of inflammatory cell infiltration. **d** The percentage of HSP70-positive cells. *p < 0.05 *versus* other groups. **e** The percentage of TUNEL-positive cells. *HSP70*, Heat shock protein 70; *TUNEL*, Terminal deoxynucleotidyl transferase-mediated dUTP nick end labeling. *p < 0.05 *versus* other groups

The esophageal squamous epithelium exhibited consistent cellular structure and morphology across all groups, maintaining a uniform thickness in the epithelial layer (60.5 ± 19.8 μm [mean ± standard deviation] *versus* 67.0 ± 40.8 μm *versus* 66.1 ± 10.0 μm *versus* 65.0 ± 50.0 μm; *p* = 0.787) (Figs. 6a and 7a). The submucosal layer showed characteristic loose connective tissue, without signs of fibrotic changes, and its vascular structures remained intact, displaying no increased vascularity. Furthermore, the thickness of this layer was consistent across the groups (68.7 ± 38.3 μm *versus* 71.3 ± 31.5 μm *versus* 57.6 ± 14.8 μm *versus* 71.3 ± 44.7 μm; *p* = 0.089) (Fig. 7b). No groups had excessive or aberrant accumulation of inflammatory cells. The levels of inflammatory cell infiltration remained statistically consistent across groups (1.2 ± 0.5 *versus* 1.2 ± 0.4 *versus* 1.0 ± 0.0 *versus* 1.4 ± 0.6; *p* = 0.506) (Fig. 7c).

### Immunohistochemical analysis

In group A, HSP70-positive cells were uniformly distributed around the circumference of the stented esophagus tissue (Fig. 6b). The percentage of these cells was significantly higher in this group than in the others (62.5 ± 23.7% *versus* 11.6 ± 7.7%, 15.6 ± 8.4%, and 8.3 ± 3.3%; all *p* < 0.001) (Fig. 7d). HSP70-positive cells spanned from the mucosal to the muscular layer. However, their count consistently decreased with increasing tissue depth, reaching its lowest in the muscularis propria. Similarly, TUNEL-positive cells in group A exhibited a uniform distribution throughout the stented esophagus and had a significantly higher percentage than the other groups (83.5 ± 14.4% *versus* 9.7 ± 6.8%, 9.6 ± 7.3%, and 6.5 ± 3.9%; all *p* < 0.001) (Figs. 6c and 7e). These cells were observed from the mucosal layer down to the muscular layer, but their number decreased as tissue depth increased, reaching its minimum in the muscularis propria. In contrast, RIP3-positive cells were not detectable across all groups (Fig. 6d).

## Discussion

It is well-recognized that hyperthermia within the temperature range of 40 to 47 °C kills cells in a reproducible, time- and temperature-dependent manner [14]. In our study, the *in vivo* surface temperature of the esophagus, stented with AuNP-integrated silicone-covered SEMS, reached this hyperthermic range upon 808 nm NIR laser irradiation. Furthermore, the AuNP-integrated silicone-covered SEMS remained intact after being subjected to a mean temperature of 47.3 °C for 2 min. This exposure translates to a CEM43 value of approximately 39.4 min, which falls within the 30 to 60 min range commonly employed in cancer therapy [13]. Additionally, staining of the stented esophagus tissue with hematoxylin and eosin and HSP70 indicated that full circumferential heating had been successfully achieved, affecting all layers from the mucosa to the muscularis propria, without causing any associated AEs.

Cell killing is one of the primary effects of hyperthermia in cancer therapy [14]. In our study, TUNEL staining of the stented esophagus tissue revealed full circumferential apoptosis across all layers, from the mucosa to the muscularis propria. However, when the same tissue samples were subjected to RIP3 staining, no evident necroptosis was observed. These findings align with expectations, as it is well-known that apoptosis can be triggered with a CEM43 of between 30 and 60 min, whereas necrotic changes usually require a CEM43 of ≥ 240 min [15]. Apart from inducing cell death, hyperthermia can also enhance the sensitivity of cancer cells to both chemo and radiation therapy [16]. This increased sensitivity may allow for the administration of these therapies at lower doses, potentially reducing side effects and improving the patient’s quality of life. Additionally, hyperthermia can stimulate systemic anticancer immune responses, potentially improving the efficacy of immunotherapy [17]. Theoretically, employing the AuNP-integrated silicone-covered SEMS for local hyperthermia can confer these benefits.

Covered SEMS placement has been the most widely used method for the palliative treatment of malignant dysphagia caused by esophageal and gastric cardia cancer [1]. However, conventional covered SEMSs only alleviate dysphagia and do not address the underlying cancer [2]. Additionally, combing external radiotherapy with SEMS placement increases the risk of severe AEs [18]. The AuNP-integrated silicone-covered SEMS, capable of mediating local hyperthermia, may simultaneously alleviate dysphagia and induce irreversible damage to cancer cells through its cell-killing effect. This approach also holds promise for use in conjunction with other anti-cancer therapies (*e.g.,* targeted and immunotherapy), as hyperthermia can enhance the delivery of anticancer drugs, improve the sensitivity of cancer cells to treatments, and trigger systemic anticancer immune responses [8].

Radio frequency ablation (Barrx; Medtronic, Minneapolis, MN, USA) has been widely utilized for eradicating Barrett’s esophagus. AEs following this procedure include stenosis, laceration, pain, hemorrhage, perforation, ulceration, dysphagia, odynophagia, and fever [19]. These AEs were reported shortly after the procedure, typically ranging from immediately to a few days later, except for stenosis, which was reported weeks to months post-procedure. Similar AEs may also arise from using the AuNP-integrated silicone-covered SEMS for local hyperthermia in the esophagus. However, the risk of these adverse events would likely be lower with the SEMS approach, which utilizes hyperthermia-range temperatures, compared to radiofrequency ablation.

Stent migration is a frequent complication of SEMS placement, occurring in approximately 23% of patients treated for malignant esophageal strictures [20]. This complication is of particular concern for patients undergoing concurrent cancer therapy, as a positive tumor response could increase the risk of stent migration [21]. It is also particularly concerning for patients receiving local cancer therapy with radioactive SEMSs, as stent migration could expose nontargeted areas to substantial radiation doses. This limitation presents a notable barrier to the broader acceptance and application of radioactive SEMSs in clinical practice. When not exposed to NIR laser, the AuNP-integrated silicone-covered SEMS acts as a conventional silicone-covered SEMS, thereby eliminating the risk of heating in non-targeted areas due to stent migration. In our study, none of the rats experiencing stent migration showed any related AEs.

This study has several notable limitations. First, we used a rat model instead of larger animal models because of the high number of animals required for this study. While rat models are commonly used in esophageal stent research for their physiological and genetic similarities to humans and ease of handling, their significant size difference from humans could pose limitations in translating these findings directly to clinical applications [22–24]. Second, we employed healthy rats rather than cancer models, which is a limitation given that cancer cells generally exhibit higher thermal sensitivity than noncancerous cells [25]. However, choosing healthy rats over cancer models was a decision driven by ethical considerations. Given hyperthermia’s established effectiveness in cancer treatment, our goal was to reduce animal distress. This is an important factor in research with the potential for clinical translation. Third, the measurements were limited to the surface temperature, which may not accurately depict the temperature within the esophageal tissue.

In conclusion, this study presented a simple and reproducible method for fabricating AuNP-integrated silicone-covered SEMSs. When exposed to 808 nm NIR laser, these SEMSs can induce local hyperthermia, thereby leading to full circumferential cell death through apoptosis in a rat esophageal model. Future studies should be oriented towards assessing this technique in larger animal models.

## Data Availability

The datasets used and/or analyzed during the current study are available from the corresponding author on reasonable request.

## Abbreviations

AEs: Adverse events
CEM43: Cumulative equivalent min at 43 °C
HSP70: Heat shock protein 70
NIR: Near-infrared
RIP3: Receptor-interacting kinase 3
SEMS: Self-expandable metal stent
TUNEL: Terminal deoxynucleotidyl transferase-mediated dUTP nick end labeling

## References

[1] Spaander MCW, van der Bogt RD, Baron TH (2021). Esophageal stenting for benign and malignant disease: European Society of Gastrointestinal Endoscopy (ESGE) Guideline - Update 2021. Endoscopy.

[2] Dai Y, Li C, Xie Y et al (2014) Interventions for dysphagia in oesophageal cancer. Cochrane Database Syst Rev 2014:Cd005048. 10.1002/14651858.CD005048.pub4 10.1002/14651858.CD005048.pub4 PMC8106614 25354795

[3] Spaander MC, Baron TH, Siersema PD (2016). Esophageal stenting for benign and malignant disease: European Society of Gastrointestinal Endoscopy (ESGE) Clinical Guideline. Endoscopy.

[4] Guo JH, Teng GJ, Zhu GY (2008). Self-expandable esophageal stent loaded with 125I seeds: initial experience in patients with advanced esophageal cancer. Radiology.

[5] Zhu HD, Guo JH, Mao AW (2014). Conventional stents versus stents loaded with (125)iodine seeds for the treatment of unresectable oesophageal cancer: a multicentre, randomised phase 3 trial. Lancet Oncol.

[6] Park JH, Park W, Cho S (2018). Nanofunctionalized stent-mediated local heat treatment for the suppression of stent-induced tissue hyperplasia. ACS Appl Mater Interfaces.

[7] Park JH, Kim MT, Kim KY (2020). Local heat treatment for suppressing gastroduodenal stent-induced tissue hyperplasia using nanofunctionalized self-expandable metallic stent in rat gastric outlet model. ACS Biomater Sci Eng.

[8] Liu Y, Crawford BM, Vo-Dinh T (2018). Gold nanoparticles-mediated photothermal therapy and immunotherapy. Immunotherapy.

[9] Kim KY, Tsauo J, Song HY, Kim PH, Park JH (2017). Self-expandable metallic stent placement for the palliation of esophageal cancer. J Korean Med Sci.

[10] Edwards DW, Laasch H-U (2015). Esophageal stents: beyond the simple stricture. Int J Gastrointest Intervent.

[11] Lee TH, Jang BS, Jung MK, Pack CG, Choi JH, Park DH (2016). Fabrication of a silver particle-integrated silicone polymer-covered metal stent against sludge and biofilm formation and stent-induced tissue inflammation. Sci Rep.

[12] Kim EY, Shin JH, Jung YY, Shin DH, Song HY (2010). A rat esophageal model to investigate stent-induced tissue hyperplasia. J Vasc Interv Radiol.

[13] Sapareto SA, Dewey WC (1984). Thermal dose determination in cancer therapy. Int J Radiat Oncol Biol Phys.

[14] Roti Roti JL (2008). Cellular responses to hyperthermia (40–46 degrees C): cell killing and molecular events. Int J Hyperthermia.

[15] Yarmolenko PS, Moon EJ, Landon C (2011). Thresholds for thermal damage to normal tissues: an update. Int J Hyperthermia.

[16] Datta NR, Ordóñez SG, Gaipl US (2015). Local hyperthermia combined with radiotherapy and-/or chemotherapy: recent advances and promises for the future. Cancer Treat Rev.

[17] Chang M, Hou Z, Wang M, Li C, Lin J (2021). Recent advances in hyperthermia therapy-based synergistic immunotherapy. Adv Mater.

[18] Nishimura Y, Nagata K, Katano S (2003). Severe complications in advanced esophageal cancer treated with radiotherapy after intubation of esophageal stents: a questionnaire survey of the Japanese Society for Esophageal Diseases. Int J Radiat Oncol Biol Phys.

[19] Dubrouskaya K, Hagenstein L, Ramai D, Adler DG (2022). Clinical adverse events and device failures for the Barrx™ radiofrequency ablation catheter system: a MAUDE database analysis. Ann Gastroenterol.

[20] Thomas S, Siddiqui AA, Taylor LJ (2019). Fully-covered esophageal stent migration rates in benign and malignant disease: a multicenter retrospective study. Endosc Int Open.

[21] Siddiqui AA, Sarkar A, Beltz S (2012). Placement of fully covered self-expandable metal stents in patients with locally advanced esophageal cancer before neoadjuvant therapy. Gastrointest Endosc.

[22] Jun EJ, Park JH, Tsauo J (2017). EW-7197, an activin-like kinase 5 inhibitor, suppresses granulation tissue after stent placement in rat esophagus. Gastrointest Endosc.

[23] Luo Y, Zhang X, Tsauo J (2021). Intragastric satiety-inducing device reduces food intake and suppresses body weight gain in a rodent model. Surg Endosc.

[24] Zhao H, Fu Y, Tsauo J (2022). Silver nanoparticle-coated self-expandable metallic stent suppresses tissue hyperplasia in a rat esophageal model. Surg Endosc.

[25] Hannon G, Tansi FL, Hilger I, Prina-Mello A (2021). The effects of localized heat on the hallmarks of cancer. Adv Therapeut.

